# Mobile Interventions and Telemedicine for Hepatitis C Elimination

**DOI:** 10.1001/jamanetworkopen.2025.55135

**Published:** 2026-01-02

**Authors:** Matthew J. Akiyama, Luis A. Gonzalez Corro, Andrew Seaman

**Affiliations:** **Author Affiliations:** Department of Medicine, Albert Einstein College of Medicine, Montefiore Medical Center, Bronx, New York (Akiyama); Department of Medicine, New York University, New York, New York (Gonzalez Corro); Department of Medicine, Division of General Internal Medicine & Geriatrics, Section of Addiction Medicine, Oregon Health & Science University, Portland (Seaman); Central City Concern, Portland, Oregon (Seaman).

The US is making progress toward a national hepatitis C virus (HCV) elimination program with the Cure Hepatitis C Act of 2025. If passed, the bipartisan bill would establish a 5-year initiative to expand access to direct-acting antiviral (DAA) therapy for vulnerable populations—such as those receiving Medicaid and in carceral facilities—through a subscription model. It would also allocate funding to strengthen public health infrastructure for screening and linkage to care.^1,2^ Estimates suggest the plan would save 24 000 lives and $13.3 billion in health expenditures over 10 years.^3^ A central question for policy makers, however, is how to ensure that expanded access translates into uptake for people who remain structurally excluded from traditional health care, such as people who use drugs.

Rural areas are disproportionally impacted by HCV due to the rise of the opioid epidemic, limited syringe services, long travel distances, and low HCV treatment adoption among rural clinicians.^4^ Friedmann et al^5^ conducted a randomized clinical trial (RCT) in rural New Hampshire and Vermont evaluating a multicomponent intervention that aimed to address key barriers to HCV elimination among rural people who use drugs. Participants with HCV and a history of injection drug use were randomized to mobile telemedicine care (MTC) for HCV, in which telemedicine was paired with mobile van HCV screening and pretreatment evaluation to bring DAA therapies directly to participants. In the control arm, or enhanced usual care (EUC), a mobile team completed screening and pretreatment evaluation and provided care navigation and facilitated referral to regional HCV-treating clinicians. The results demonstrated that 57.3% of those in MTC initiated DAA therapy compared with 26.7% in EUC, and 37.3% of MTC patients achieved documented viral clearance vs 18.7% in EUC.

Mobile interventions overcome barriers to engagement in traditional health care settings by bringing health care and other services directly to people in the community (eg, near syringe service programs or encampments for people who are unhoused).^6,7^ Many mobile HCV care models colocate HCV testing, evaluation, and treatment, a process often requiring multiple encounters in traditional health care settings. Delivering HCV care in community spaces where people who use drugs are located—by necessity or by choice—alleviates structural and social barriers such as transportation, fragmented referral pathways, and stigma. Mobile interventions demonstrate how meeting people where they are can meaningfully improve access and strengthen the HCV care cascade.

Central to the intervention in the study by Friedmann et al^5^ was integrating telemedicine into the mobile van. Telehealth interventions have been instrumental to expanding HCV treatment access in the US for nearly 2 decades. Extension for Health Outcomes (EHCO), founded in 2004, introduced a telementoring model that moved HCV treatment from specialist clinics into primary care, beginning in rural New Mexico. The ECHO model has since increased the number of HCV-treating clinicians nationwide,^8^ yet many people with HCV remain unengaged in primary care. Direct telemedicine represents a critical tool to reach these individuals where they are and initiate treatment outside traditional health care settings. Telemedicine has been used effectively among people who use drugs, demonstrating high HCV cure rates. In an RCT of opioid treatment program (OTP)–integrated telemedicine in New York, patients who received an initial telemedicine encounter facilitated on-site at the OTP had significantly higher HCV cure rates compared with off-site referral to HCV-treating clinicians.^9^ In another RCT, the Peer TeleHCV study, rural peer-assisted telemedicine led to 6 times higher treatment initiation rates and 4 times higher cure rates compared with peer-assisted referral to local HCV-treating clinicians,^10^ underscoring the importance of community-based, assisted telemedicine in rural HCV elimination efforts.

Friedmann et al^5^ built on this literature with, to our knowledge, the first randomized evaluation of a test-to-cure facilitated telemedicine HCV treatment model based out of a mobile, community-facing care platform. Their finding that mobile telemedicine doubled both treatment initiation and cure rates among rural people who use drugs relative to enhanced referral suggests that navigation support alone is not sufficient to allow this population to access brick-and-mortar health care. In aggregate, this emerging literature shows that telemedicine—whether facilitated through OTPs, community-based peer interventions, or mobile vans—is a critical tool for lowering barriers to HCV treatment among people who use drugs. Beyond facilitated telemedicine, however, wide ranges in treatment initiation and viral clearance rates make determining best practices from these multicomponent interventions difficult to interpret. These studies span urban vs rural environments with varied intervention components (eg, mobile health, syringe services, peer navigation, adherence support, medication storage lockers, facilitation with mail-order pharmacy logistics, and more).^6,7,9,10^ Additionally, disengagement from care after diagnosis is common, with several studies (including that by Friedmann et al^5^) demonstrating high rates of loss to follow-up. While lower-barrier care increases treatment uptake, continued support is needed to ensure engagement in the HCV care cascade through confirmation of cure and, if needed, assessment for reinfection. The optimal combination of support for specific environments requires more research. Peers have been shown to be effective in engaging rural people who inject drugs,^10^ but the optimal peer navigator roles, including required training and intensity of engagement, have not been well defined. Despite these needs for additional data, many of the most prominent barriers to care for rural people who use drugs are revisited with every step of the care cascade, and it is increasingly clear that removing steps through co-location of services is paramount.

We are at a critical juncture in the fight against HCV in the US. The Cure Hepatitis C Act could enhance the nation’s HCV elimination efforts, if passed. Yet, existing models of testing and treating rural people who use drugs and who rely on traditional health care systems are insufficient to reach HCV elimination targets. By demonstrating that a mobile, telemedicine-delivered treatment model can outperform enhanced navigation alone in rural settings, Friedmann et al^5^ provide important evidence that decentralizing care and expanding telemedicine access is essential for addressing structural barriers to HCV treatment among rural people who inject drugs. Given that trials have combined multicomponent interventions, further research may be necessary to determine the relative value of each component and how such components fare in specific settings (eg, urban vs rural). In addition, future studies should evaluate how mobile models can integrate sustained peer support, longitudinal harm-reduction engagement, and reinfection prevention strategies to maintain long-term treatment success. Scaling these approaches will require investment in infrastructure, workforce capacity, and reimbursement pathways that recognize mobile telemedicine as a core element of HCV elimination, not an adjunct. If scaled with intention, decentralized models could redefine what equitable HCV care looks like in the US. Now is the moment to move from possibility to practice.
